# Analysis of Depression and Anxiety in Patients With Tinnitus: A Focus on Specific Age Groups and Sex‐Related Differences

**DOI:** 10.1155/da/9170627

**Published:** 2026-05-12

**Authors:** Jing Zhou, Yuehong Liu, Hongbo Xie, Siyi Yang, Yun Jiang, Zhao Han

**Affiliations:** ^1^ Department of Otorhinolaryngology–Head and Neck Surgery, Huadong Hospital Affiliated Fudan University, Shanghai, 200040, China, fudan.edu.cn

**Keywords:** age-stratified analysis, anxiety, chronic subjective tinnitus, comorbidities, depression, insomnia, quality of life, sex differences

## Abstract

**Objective:**

To investigate the relationships between anxiety, depression, and insomnia and tinnitus severity among high‐functioning employed adults, stratified by age and gender, and to describe age‐ and gender‐specific patterns of prevalence and symptom burden in order to support a biopsychosocial framework for understanding tinnitus onset, progression, and clinical management.

**Methods:**

An analysis was performed on data from 745 patients with chronic subjective tinnitus, aged 25–60 years, to assess levels of anxiety (HADS‐A), depression (HADS‐D), insomnia (ISI), and the perceived severity of tinnitus (THI and VAS).

**Results:**

The findings showed that anxiety was significantly correlated with the tinnitus Handicap Inventory in younger patients, particularly in men (*r* = 0.611, *p*  < 0.001) and women (*r* = 0.577, *p*  < 0.001) aged 25–40 years. In women aged 51–60 years, depression demonstrated the strongest association with tinnitus (*r* = 0.545, *p*  < 0.001). In the overall cohort, insomnia exhibited a weak but statistically significant correlation with tinnitus (*r* ≤ 0.3). Additionally, women were more likely to link tinnitus loudness to reduced quality of life, especially in the 25–40 (*r* = 0.722, *p*  < 0.01) and 51–60 (*r* = 0.689, *p*  < 0.01) age groups.

**Conclusion:**

The findings indicate that anxiety in younger patients is closely related to tinnitus, whereas depressive symptoms are more prominent among middle‐aged and older women. These variations indicate that patients with tinnitus may present different psychological profiles across gender and age groups. Future research may further explore the underlying pathophysiological mechanisms of tinnitus and examine its progression through long‐term follow‐up to improve understanding of its clinical heterogeneity.

## 1. Introduction

Chronic subjective tinnitus is a common auditory condition in which patients perceive persistent phantom sounds without any external source, leading to considerable reductions in quality of life (QOL) [1]. Individuals with chronic tinnitus often experience comorbid psychological issues, including anxiety, depression, and sleep disturbances, which negatively affect personal well‐being and contribute to significant societal burdens [2]. Individuals with chronic tinnitus face a higher risk of developing mental health conditions such as anxiety, depression, and insomnia, which can lower life satisfaction and impose high economic costs [3]. Although previous research has explored the relationships between tinnitus and anxiety, as well as between depression and insomnia, further studies are needed to clarify the complex interactions among these psychological factors and chronic tinnitus, with the goal of enhancing patient outcomes more broadly [4].

Clinical observations show that individuals in this age group, often in the peak of their professional careers, are especially susceptible to increased life stress after the onset of tinnitus. Since tinnitus involves complex and systemic processes, examining only otological abnormalities is insufficient for a full understanding, as broader systemic and psychosocial factors are commonly implicated [4]. Although earlier studies have explored the psychological effects of tinnitus and its impact on QOL, few clinical investigations have systematically evaluated age‐related differences in these outcomes, especially using stratified analyses across the 25–60 year age range. To more accurately assess tinnitus‐related distress and its psychological associations, this study systematically investigated the relationships between tinnitus severity and symptoms of comorbid anxiety, depression, and sleep disturbances, including further analyses stratified by gender and age group [5]. Measuring these psychological factors can help clinicians tailor treatment strategies, ultimately enhancing patient prognosis and QOL.

Chronic subjective tinnitus is a complex condition that rarely occurs in isolation. Although this study emphasizes its strong association with psychological distress, including anxiety and depression, increasing evidence also points to its association with various somatic comorbidities. For example, a recent comprehensive analysis highlighted significant links between tinnitus and cardiovascular conditions such as atherosclerosis and dyslipidemia, as well as metabolic and autoimmune disorders, emphasizing the systemic aspects of tinnitus. These findings support the need for a holistic clinical evaluation that goes beyond audiological and psychological factors [6]. Therefore, this study aims to investigate the relationships between emotional distress, sleep disturbances, and subjective tinnitus severity and to determine whether these associations differ across demographic groups, providing a foundation for more comprehensive clinical and classification approaches.

## 2. Materials and Methods

### 2.1. Patients

This study included patients aged 25–60 years with chronic tinnitus who attended the otolaryngology outpatient clinic of our hospital between January 2018 and August 2024. All participants reported tinnitus as their primary symptom. Each patient underwent a thorough examination and received follow‐up care. This study was approved by the Ethics Committee of Huadong Hospital Affiliated with Fudan University on January 20, 2024 (Ethics Committee of Huadong Hospital Affiliated to Fudan University: 20240120). Written informed consent was obtained from all participants before treatment. Inclusion criteria: 1. Age between 25 and 60 years; 2. Presence of subjective tinnitus for at least 1 year; 3. Ability to independently complete study questionnaires (Tinnitus Handicap Inventory [THI], Tinnitus Loudness Visual Analog Scale [VAS], Hospital Anxiety and Depression Scale [HADS‐A/D], and Insomnia Severity Index [ISI]) and participate in all required assessments; 4. No evident organic lesions on audiological examination and no hearing loss that significantly impairs daily communication. Exclusion criteria: 1. Severe neurological or psychiatric disorders, including severe anxiety, major depressive disorder, schizophrenia, or similar conditions; 2. Receipt of tinnitus treatment within the past 3 months, including medication, hearing aids, or other sound therapies; 3. Presence of objective tinnitus, such as vascular tinnitus or middle ear myoclonus; 4. Impaired language comprehension, dyslexia, pregnancy, or breastfeeding; 5. Organic ear diseases, including sudden sensorineural hearing loss (defined as a loss of ≥30 dB over at least three consecutive frequencies within 72 h), Ménière’s disease, otitis media, or otosclerosis; only acute sudden sensorineural hearing loss cases were excluded, while tinnitus linked to other chronic or stable sensorineural hearing loss was included as primary tinnitus; 6. Inability to complete follow‐up examinations or incomplete questionnaire responses. Age stratification: participants were categorized into three groups based on sociological roles and biological changes: 25–40 years, 41–50 years, and 51–60 years. Data collection and analyses: all participants met the inclusion criteria, with complete documentation of medical history, tinnitus characteristics (including duration, pitch frequency, and intensity), psychiatric status, and sleep assessment. A cross‐sectional study design was applied to perform baseline statistical analyses and to examine the associations between tinnitus, psychological symptoms, and sleep disturbances across the different age groups.

### 2.2. Audiological and Medical Assessment

All participants underwent a thorough otological and audiological evaluation performed by qualified otolaryngologists and audiologists. The assessment included otoscopic examination, pure‐tone audiometry with air‐ and bone‐conduction thresholds measured at standard frequencies (0.25–8 kHz), speech audiometry, and immittance testing, including tympanometry and acoustic reflex measurements. All audiological assessments were conducted in a sound‐treated booth using calibrated clinical audiometric equipment following established clinical guidelines. Tinnitus psychoacoustic evaluations, including pitch and loudness matching, were performed using standard audiometric methods to characterize the perceptual features of tinnitus. To exclude secondary causes of tinnitus, especially retrocochlear pathology, patients with suspicious clinical signs received additional neurophysiological and imaging evaluations, including auditory brainstem response (ABR) testing and/or magnetic resonance imaging (MRI) of the internal auditory canal and cerebellopontine angle. Cases of secondary tinnitus caused by identifiable structural or retrocochlear lesions were excluded based on these results.

### 2.3. Clinical Assessment and Data Collection

In this study, an otolaryngologist established the initial diagnosis of tinnitus using medical history, physical examination, and audiological evaluation. Demographic information (age and sex) and tinnitus characteristics (loudness and frequency) were recorded. Clinical variables, including tinnitus duration, pitch, loudness, and age, were documented for stratified analyses. These data were used to examine potential differences in the relationships between tinnitus severity, psychological distress, and sleep disturbances across subgroups, supporting more individualized clinical assessment and management.

### 2.4. Questionnaires and Outcome Measures

Tinnitus severity and symptoms of anxiety, depression, and sleep disturbances were evaluated using validated self‐report instruments, including the THI, the VAS, HADS‐A and HADS‐D, and ISI. All questionnaires used in this study were validated in the primary language of the participants and have shown good reliability and validity in previous clinical research. The THI is a tinnitus‐specific questionnaire containing 25 items divided into three domains: functional, emotional, and catastrophic. These domains evaluate the effects of tinnitus on daily activities, emotional well‐being, and catastrophic responses, respectively [7]. Total THI scores range from 0 to 100, with higher scores reflecting greater tinnitus‐related handicap. Scores are typically classified as slight (0–16), mild (18–36), moderate (38–56), severe (58–76), and catastrophic (78–100). The VAS was used to measure the subjective perception of tinnitus loudness on a 0–10 scale, where higher scores indicate louder perceived tinnitus [7]. Psychological distress was assessed using the HADS, which includes two 7‐item subscales measuring anxiety (HADS‐A) and depression (HADS‐D), each scored from 0 to 21 [8]. Scores of 0–7 indicate no clinically significant symptoms, 8–10 suggest possible anxiety or depression, and 11–21 indicate a definite presence of anxiety or depression. Sleep disturbance was evaluated using the ISI, a 7‐item questionnaire that measures the severity of insomnia symptoms, with total scores ranging from 0 to 28 [9]. Scores of 0–7 indicate no clinically significant insomnia, 8–14 indicate subthreshold insomnia, 15–21 indicate moderate insomnia, and 22–28 indicate severe insomnia.

### 2.5. Statistical Methods

The Shapiro–Wilk test was used to assess the normality of continuous variables. Spearman correlation analysis was performed to examine the relationships among THI, VAS, HADS‐A/D, and ISI, allowing assessment of the associations between tinnitus severity, psychological symptoms, and sleep disturbances. To explore potential differences across age and sex groups, one‐way analysis of variance (ANOVA) was used for variables with a normal distribution, while the Kruskal–Wallis *H* test was applied for non‐normally distributed variables. Stratified analyses were conducted to assess the effects of age, tinnitus duration, and tinnitus frequency on symptom presentation. Post hoc multiple comparisons were performed when appropriate. All statistical analyses were conducted using SPSS version 26.0 (IBM Corp., Armonk, NY, USA), and a two‐tailed *p* value of less than 0.05 was considered statistically significant.

## 3. Results

A total of 745 outpatients aged 25–60 years with chronic subjective tinnitus were included in the study. The patient characteristics are shown in Table 1. Among them, 382 were male (51.3%), and 363 were female (48.7%). Age was approximately normally distributed, with a median of 40.5 years (IQR: 34–48) for males and 40 years (IQR: 35–47) for females. There was no significant difference in age distribution between the two groups (*P* = 0.962). All participants were divided into three age groups (25–40 years, 41–50 years, and 51–60 years) for age‐stratified analyses to ensure statistical comparability. The distribution of males and females across the age groups was as follows: in the 25–40 years group, 191 males (25.6%) and 191 females (25.6%); in the 41–50 years group, 117 males (15.7%) and 107 females (14.4%); in the 51–60 years group, 74 males (9.9%) and 65 females (8.7%). The gender distribution across age groups did not differ significantly (*P* = 0.761), indicating that the proportion of males and females was similar across all age categories.

**Table 1:** Baseline characteristics of patients with chronic subjective tinnitus stratified by sex.

Characteristics	Male	Female	*p* value
*n*	382	363	—
Age, median (IQR)	40.5 (34, 48)	40 (35, 47)	0.962
Group, *n* (%)	—	—	0.761
25–40	191 (25.6%)	191 (25.6%)	1.00
41–50	117 (15.7%)	107 (14.4%)	0.47
51–60	74 (9.9%)	65 (8.7%)	0.42

*Note*: Data are presented as median (interquartile range, IQR) or *n* (%), as appropriate. Between‐group comparisons were conducted using the Mann–Whitney *U* test for continuous variables and the chi‐square test or Fisher’s exact test for categorical variables, as appropriate. A two‐tailed *p* value of less than 0.05 was considered statistically significant.

Abbreviation: IQR, interquartile range.

**Figure 1:**
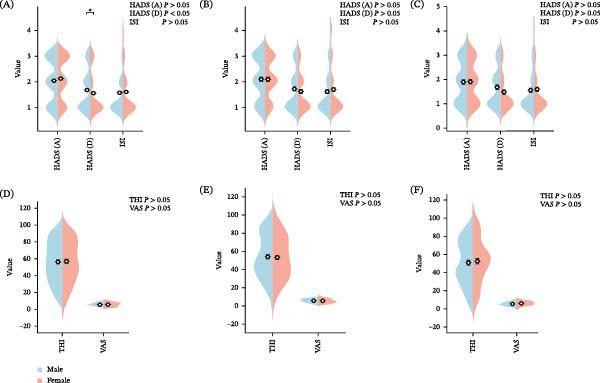
A–C HADS (A), HADS (D), and ISI scale scores for males and females in the 25–40, 41–50, and 51–60 age groups; D–F THI and VAS scale scores for males and females in the 25–40, 41–50, and 51–60 age groups. Note: Stratified analysis of HADS‐A, HADS‐D, ISI, THI, and VAS scores by age group and sex. Abbreviations: HADS‐A, hospital anxiety and depression scale‐anxiety; HADS‐D, hospital anxiety and depression scale‐depression; ISI, insomnia severity index; THI, tinnitus handicap inventory; VAS, visual analog scale.

In this study, a gender‐specific analysis was conducted to examine the subjective impact of anxiety, depression, insomnia, and tinnitus in patients aged 25–60 years. As illustrated in Figure 1, HADS‐A (anxiety) scores did not differ significantly between men and women across any age group (*p*  > 0.05), indicating that anxiety levels may be influenced more by tinnitus than by gender. In the 25–40 years (A/D) age group, women had significantly higher HADS‐D (depression) scores than men (*p*  < 0.05), indicating that younger women with tinnitus may be more vulnerable to depression. In contrast, in the 41–50 years (B/E) and 51–60 years (C/F) age groups, no significant gender differences were observed (*p*  > 0.05), suggesting that psychological adaptability may improve with age. Furthermore, ISI scores did not differ significantly between men and women in any age group (*p* = 0.47), indicating that the impact of tinnitus‐related sleep disturbances was similar across genders. Regarding the subjective impact of tinnitus (THI) and perceived tinnitus loudness (VAS), no significant differences were observed between the men and women (*p*  > 0.05), indicating that the effect and perceived severity of tinnitus on daily life are similar across sexes.

**Figure 2:**
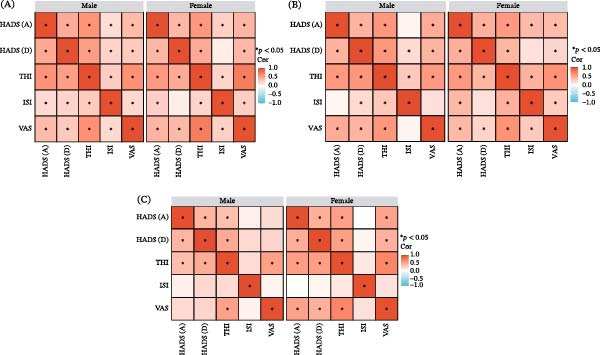
Analysis of correlation between the 5 scales for men and women aged 25–40, 41–50, 51–60. Note: Correlation matrices of THI, HADS‐A, HADS‐D, ISI, and VAS scores for different age groups: (A) young group (18–39 years), (B) middle‐aged group (40–59 years), (C) older group (≥60 years). Abbreviations: HADS‐A, hospital anxiety and depression scale (anxiety); HADS‐D, hospital anxiety and depression scale (depression); ISI, insomnia severity index; THI, tinnitus handicap inventory; VAS, visual analog scale.

Figure 2 shows a correlation analysis of 745 patients aged 25–60 years with chronic subjective tinnitus, examining the relationships between anxiety, depression, insomnia, and tinnitus severity and highlighting psychological patterns across different age and sex groups. Overall, Figure 2 demonstrates moderate to strong positive correlations, suggesting a significant interaction between emotional disorders, sleep disturbances, and the subjective burden of symptoms in patients with chronic tinnitus. The strongest associations were observed in the older age group (Figure 2C).

**Figure 3:**
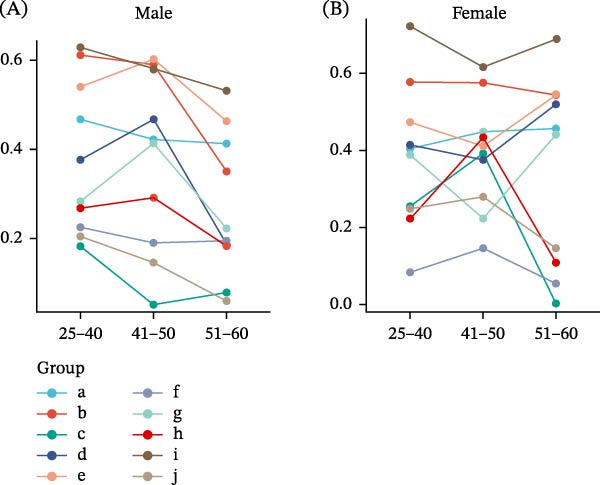
(A) Male and (B) Female. (a) HADS‐A and HADS‐D, (b) HADS‐A and THI, (c) HADS‐A and ISI, (d) HADS‐A and VAS, (e) HADS‐D and THI, (f) HADS‐D and ISI, (g) HADS‐D and VAS, (h) THI and ISI, (i) THI and VAS, and (j) ISI and VAS.

In women aged 51 years and older, the linear relationships between emotional disturbances, sleep problems, and the perceived burden of tinnitus were weaker than in the younger age groups. This suggests a relative separation between psychological symptoms and perceived tinnitus severity in this subgroup. The weakening of correlations, particularly those involving anxiety, depression, and insomnia scores, suggests that tinnitus‐related distress in older women may be influenced by additional nonpsychological or age‐related factors. In contrast, men in all age groups exhibited a relatively consistent pattern of correlations, with similar effect sizes across age strata. This indicates a stable association between psychological symptoms and tinnitus, with emotional distress, sleep disturbances, and tinnitus burden remaining closely linked throughout adulthood. Anxiety (HADS‐A) was significantly positively correlated with the THI in men (*r* = 0.611) and women (*r* = 0.577) aged 25–40 years. This correlation was weaker in men aged 51–60 years (*r* = 0.35) (Table 2 and Figure 3). The association between depression (HADS‐D) and tinnitus was strongest in women aged 51–60 years (*r* = 0.545), compared with men in the same age group (*r* = 0.463), indicating that menopausal women may be more vulnerable to depression due to physiological and psychological changes. In contrast, the overall correlation between insomnia (ISI) and tinnitus was low (*r* ≤ 0.3), suggesting that insomnia may act more as a secondary symptom rather than a primary driver of tinnitus. Additionally, the correlation between tinnitus loudness (VAS) and tinnitus impact (THI) was stronger in women aged 25–40 years (*r* = 0.722) and 51–60 years (*r* = 0.689) than in men (highest *r* = 0.628), suggesting that women are more likely to link tinnitus loudness to reduced QOL (Table 3 and Figure 3).

**Table 2:** Correlation coefficients among the five scales for males aged 25–40, 41–50, and 51–60 years.

Age (male)	HADS (A) and HADS (D)	HADS (A) and THI	HADS (A) and ISI	HADS (A) and VAS	HADS (D) and THI	HADS (D) and ISI	HADS (D) and VAS	THI and ISI	THI and VAS	ISI and VAS
25–40	0.466 ^∗∗∗^	0.611 ^∗∗∗^	0.182 ^∗∗∗^	0.376 ^∗∗^	0.539 ^∗∗∗^	0.225 ^∗∗^	0.284 ^∗^	0.268 ^∗∗∗^	0.628 ^∗∗∗^	0.204 ^∗∗∗^
41–50	0.422 ^∗∗^	0.589 ^∗∗∗^	0.052 ^∗∗^	0.467 ^∗∗∗^	0.602 ^∗∗∗^	0.189 ^∗∗∗^	0.411 ^∗∗^	0.291 ^∗^	0.580 ^∗∗∗^	0.146 ^∗∗^
51–60	0.412 ^∗∗^	0.350 ^∗^	0.079 ^∗∗^	0.188 ^∗∗^	0.463 ^∗∗∗^	0.196 ^∗∗^	0.222 ^∗∗^	0.183 ^∗∗^	0.531 ^∗∗∗^	0.060 ^∗∗∗^

Abbreviations: HADS‐A, hospital anxiety and depression scale (anxiety); HADS‐D, hospital anxiety and depression scale (depression); ISI, insomnia severity index; THI, tinnitus handicap inventory; VAS, visual analog scale.

^∗^ *p*  < 0.05.

^∗∗^ *p*  < 0.01.

^∗∗∗^ *p*  < 0.001.

**Table 3:** Correlation coefficients among the five scales for females aged 25–40, 41−50, and 51–60 years.

Age (female)	HADS (A) and HADS (D)	HADS (A) and THI	HADS (A) and ISI	HADS (A) and VAS	HADS (D) and THI	HADS (D) and ISI	HADS (D) and VAS	THI and ISI	THI and VAS	ISI and VAS
25–40	0.404 ^∗∗^	0.577 ^∗∗∗^	0.255 ^∗∗∗^	0.414 ^∗∗^	0.473 ^∗∗∗^	0.083 ^∗∗∗^	0.388 ^∗∗^	0.223 ^∗∗∗^	0.722 ^∗∗∗^	0.248 ^∗∗∗^
41–50	0.448 ^∗∗^	0.575 ^∗∗∗^	0.392 ^∗∗^	0.376 ^∗∗^	0.410 ^∗∗^	0.146 ^∗∗^	0.221 ^∗∗∗^	0.433 ^∗∗^	0.616 ^∗∗∗^	0.279 ^∗∗^
51–60	0.455 ^∗∗∗^	0.544 ^∗∗∗^	0.002 ^∗∗^	0.519 ^∗∗∗^	0.545 ^∗∗∗^	0.053 ^∗∗^	0.440 ^∗∗^	0.108 ^∗∗^	0.689 ^∗∗∗^	0.146 ^∗∗^

Abbreviations: HADS‐A, hospital anxiety and depression scale (anxiety); HADS‐D, hospital anxiety and depression scale (depression); ISI, insomnia severity index; THI, tinnitus handicap inventory; VAS, visual analog scale.

^∗^ *p*  < 0.05.

^∗∗^ *p*  < 0.01.

^∗∗∗^ *p*  < 0.001.

## 4. Discussion

This study investigated the relationships between tinnitus‐related handicap and perceived loudness (THI/VAS) and symptoms of anxiety, depression, and insomnia (HADS/ISI) in a working‐age outpatient cohort aged 25–60 years, divided into age groups of 25–40, 41–50, and 51–60 years. The primary goal was not to confirm the co‐occurrence of tinnitus and psychological distress, but to examine whether the strength and patterns of these associations vary by age and sex in a large working‐age population. Our findings revealed clear age‐ and sex‐specific differences. Anxiety showed the strongest correlation with perceived tinnitus loudness in younger adults. Depressive symptoms were more pronounced in middle‐aged and older women. The impact of insomnia on tinnitus was relatively modest.

Chronic subjective tinnitus is a prevalent auditory condition characterized by the perception of sound without any external acoustic or electrical stimulus [10]. It often occurs alongside hearing loss, noise exposure, or certain medications, though its cause remains unknown in many cases. Tinnitus can significantly reduce QOL, and [5] beyond the perception of sound, its disruption of daily activities is a major contributor to patient distress. Recent studies show that tinnitus can directly affect key communication abilities, with higher tinnitus intensity strongly associated with reduced speech comprehension, highlighting a significant functional burden for affected individuals [11]. A recent study examined hematological factors and found that lower hemoglobin and platelet levels were significant predictors of tinnitus severity and bilaterality, suggesting possible involvement of oxygen transport and inflammatory mechanisms [12]. Numerous studies have consistently shown that comorbid psychological conditions, such as anxiety, depression, and insomnia, are common in patients with tinnitus and contribute to additional clinical and societal burdens [13]. These findings support an integrative, stratified approach to understanding tinnitus rather than focusing solely on otological aspects.

Our results are consistent with epidemiological data showing that the prevalence of tinnitus increases with age [14] and with studies reporting that bothersome tinnitus and its functional impact are more common after midlife [14]. We also confirm the well‐established finding that tinnitus severity is associated with emotional distress and sleep disturbances [13, 15]. The novel contribution of this study lies in identifying age‐ and sex‐specific association patterns within a large working‐age cohort, rather than reporting only pooled correlations. Practically, these patterns indicate that the strongest predictor of tinnitus loudness (VAS) may differ from the main predictor of tinnitus handicap (THI) and that these relationships vary across subgroups.

In women, our findings are consistent with previous reports showing greater tinnitus‐related functional impact, as indicated by higher THI scores, and suggest that sex‐related factors may influence symptom burden [16]. Neuroimaging studies also indicate sex differences in tinnitus‐related brain networks, including functional connections between auditory regions and the anterior cingulate cortex, with women exhibiting higher emotional reactivity [17]. Collectively, these findings support the possibility of sex‐related differences in how tinnitus distress is expressed, without pointing to a single underlying mechanism.

Although our effect sizes and patterns do not exactly replicate those of previous studies, several plausible, nonexclusive explanations exist. First, the sampling context is important. Outpatient cohorts may include individuals who seek care because of interference and distress, which can amplify observed associations [5]. Second, differences in the constructs measured are relevant. THI assesses perceived handicap and the emotional and functional impact of tinnitus, while VAS measures perceived loudness. These related but distinct measures may show different associations with anxiety, depression, and insomnia across subgroups. Third, cohort characteristics and study timing are important. Working‐age populations may experience unique stressors, caregiving responsibilities, and occupational demands that are less common in studies focused on older, retired populations [14, 18]. These factors offer a cautious explanation for differences between studies, without making mechanistic inferences based solely on questionnaire data [14, 18].

A key observation was the relatively modest correlation between insomnia and tinnitus measures across age groups. Although insomnia is commonly reported in patients with tinnitus and remains clinically important, it showed weaker associations than anxiety and depression in our cohort. This finding aligns with previous research indicating that tinnitus often co‐occurs with psychiatric symptoms and that affective symptoms may be more closely linked to tinnitus‐related distress than sleep disturbances alone [15]. Recent studies also emphasize strong associations between tinnitus burden and anxiety or depression [15]. From a psychiatric systems perspective, ongoing tinnitus percepts may not be effectively regulated by emotional control circuits, leading to distress in which sleep disturbances may emerge as secondary or collateral symptoms [19]. Clinically, these findings support the need for routine screening of anxiety and depression alongside sleep evaluation, rather than assuming that insomnia is the primary correlate of tinnitus burden.

We also found age‐related changes in the patterns of association. In younger adults, anxiety was more strongly linked to perceived tinnitus loudness, while in women aged 41–50 and 51–60 years, depressive symptoms showed a stronger association with tinnitus handicap. These subgroup‐specific patterns provide useful clinical insights, indicating when tinnitus‐related loudness may be closely connected to arousal or anxiety and when handicap‐related distress may correspond more with low mood. Although this does not suggest separate causal mechanisms, it can help guide assessment priorities and referral decisions.

We conceptualized tinnitus as a percept or symptom that can become distressing and disabling in some individuals, rather than as a uniform “disorder” or “illness.” Recent research increasingly frames tinnitus burden as involving both emotional and sensory dysregulation, rather than solely auditory pathology [20]. Clinical observations also highlight high rates of comorbidity with anxiety, depression, and insomnia [21]. In younger patients, limited top‐down emotional regulation may enhance the link between anxiety and tinnitus salience [20], supporting the clinical rationale for interventions tailored to individual emotional and cognitive profiles [22].

We interpret our results within a transdiagnostic distress framework. Anxiety, depression, and insomnia share common features, including negative affectivity and hyperarousal [13], and are frequently comorbid rather than fully separable in clinical practice. Thus, the differing correlations observed in our study should not be taken as evidence of independent constructs or strictly separate treatment pathways. Rather, they suggest that overlapping dimensions of distress may relate differently to perceived tinnitus loudness (VAS) versus functional and emotional handicap (THI), with these relationships varying across subgroups.

Biological factors related to sex and age may influence these associations. Hormonal changes during perimenopause can alter hearing sensitivity even in the absence of measurable audiometric loss [23]. Menopause is also linked to a higher susceptibility to anxiety and depressive symptoms [24]. Declining estrogen levels may impact vestibular and cochlear function as well as neurotransmitter regulation [25], and fluctuations in estrogen have been suggested to affect cortical inhibitory circuits [25]. Previous research has also indicated that reduced estrogen‐related regulation of emotion centers, such as the amygdala, may heighten emotional responses [26]. Additionally, stress history and past trauma can influence both tinnitus perception and associated distress [27]. The psychosocial context can also differ by life stage. Women aged 25–40 years often manage combined work and caregiving responsibilities, whereas women aged 51–60 years may undergo transitions that increase somatic awareness and the attribution of symptoms to emotional factors [27]. Overall, these findings support an interaction model in which tinnitus perception, distress dimensions, hormonal factors, and psychosocial demands together influence the overall symptom burden [17, 20–22].

Several limitations should be noted. First, the cross‐sectional design prevents conclusions about causality or the direction of effects. The observed associations cannot separate bottom‐up sensory influences from top‐down emotional and cognitive processes. Second, the primary measures were self‐reported questionnaires, which may be subject to reporting bias. Third, we did not directly assess important factors such as detailed hearing status, cognitive appraisal patterns, or objective stress exposure, limiting mechanistic interpretation. Fourth, the study was conducted at a single outpatient center, which may reduce generalizability. Fifth, the extended recruitment period could have introduced contextual influences, including COVID‐19–related effects on distress reporting. Future research should employ longitudinal, multicenter designs and incorporate audiometric, psychophysical, and cognitive–behavioral measures to test specific models and clarify causal pathways [28]. Additionally, targeted intervention studies could investigate whether distress profiles within specific subgroups predict treatment outcomes, including approaches informed by psychological interventions such as CBT [29].

## 5. Conclusion

This study revealed clear age‐ and sex‐specific differences in the relationships among emotional states, sleep disturbances, and subjective tinnitus perception. Anxiety was more strongly linked to tinnitus loudness in younger patients, while depressive symptoms were more pronounced in middle‐aged and older women. The impact of insomnia was comparatively modest. By examining subgroup‐specific associations rather than pooled correlations, these findings highlight how different dimensions of distress relate to distinct aspects of tinnitus and support a more nuanced, transdiagnostic approach to assessment in clinical practice, rather than assuming rigidly separate treatment pathways [30, 31]. The results should be interpreted considering the cross‐sectional design, reliance on self‐reported measures, and single‐center sample [32, 33]. Despite these limitations, this study provides a clinically relevant framework for more personalized tinnitus management and prioritization of psychological targets, and it underscores the need for future longitudinal, multicenter studies incorporating objective measures [34].

## Author Contributions

**Jing Zhou and Yuehong Liu**: methodology and data collection, writing – original draft. **Hongbo Xie, Siyi Yang, and Yun Jiang:** formal analysis and data curation. **Zhao Han**: conceptualization, writing – review and editing.

## Funding

This work was supported by the Shanghai Municipal Commission of Science and Technology Fund (Grant 20Y11902300) by the China government.

## Disclosure

All authors have read and agreed to the final version of the manuscript. The funders had no role in study design, data collection, data analysis, data interpretation, or writing of the report. This manuscript acknowledges that no individuals or third‐party services, who are not listed as authors or mentioned in the acknowledgments section, have participated in the research or manuscript preparation.

## Conflicts of Interest

The authors declare no conflicts of interest.

## Data Availability

Parts of the data analyzed during this study are included in the published article. Full data sets generated and analyzed during the study are available from the first author or the corresponding author on reasonable request.
